# Built environment interventions aimed at improving physical activity levels in rural Ontario health units: a descriptive qualitative study

**DOI:** 10.1186/s12889-015-1786-2

**Published:** 2015-05-03

**Authors:** Cara-Lee Coghill, Ruta K Valaitis, John D Eyles

**Affiliations:** 285 Briarhill Road, Woodstock, N4S 7T4 Ontario, Canada; School of Nursing, Faculty of Health Sciences, McMaster University, Hamilton, , L8S 4K1, , Ontario Canada; School of Geography & Earth Sciences, Faculty of Science, McMaster University, Hamilton, , L8S 4K1, , Ontario Canada; Centre for Health Policy, School of Public Health, University of Witwatersrand, Johannesburg, South Africa

**Keywords:** Built environment, Physical activity, Public health, Rural, Interventions

## Abstract

**Background:**

Few studies to date have explored the relationship between the built environment and physical activity specifically in rural settings. The Ontario Public Health Standards policies mandate that health units in Ontario address the built environment; however, it is unclear how public health practitioners are integrating the built environment into public health interventions aimed at improving physical activity in chronic disease prevention programs.

**Methods:**

This descriptive qualitative study explored interventions that have or are being implemented which address the built environment specifically related to physical activity in rural Ontario health units, and the impact of these interventions. Data were collected through twelve in-depth semi-structured interviews with rural public health practitioners and managers representing 12 of 13 health units serving rural communities. Key themes were identified using qualitative content analysis.

**Results:**

Themes that emerged regarding the types of interventions that health units are employing included: Engagement with policy work at a municipal level; building and working with community partners, committees and coalitions; gathering and providing evidence; developing and implementing programs; and social marketing and awareness raising. Evaluation of interventions to date has been limited.

**Conclusions:**

Public health interventions, and their evaluations, are complex. Health units who serve large rural populations in Ontario are engaging in numerous activities to address physical activity levels. There is a need to further evaluate the impact of these interventions on population health.

**Electronic supplementary material:**

The online version of this article (doi:10.1186/s12889-015-1786-2) contains supplementary material, which is available to authorized users.

## Background

Over the past two decades, obesity rates have nearly doubled and physical activity levels have decreased among Canadian youth and adults causing concern among public health practitioners [1]. According to measured height and weight data from the 2008 Canadian Community Health Survey (CCHS), 62.1% of adult Canadians are overweight or obese, with 25.4% classified as obese [1]. The Community Health Measures Survey data from 2007–2009 indicates that only 15% of Canadian adults and 7% of children and youth aged 6 to 19 years of age meet the recommended physical activity guidelines [2,3]. In 2009–2010, 45.2% of Canadians reported that they were inactive, with physical activity decreasing with age [4]. In rural settings, residents have been found to have significantly higher obesity rates and lower physical activity rates than their urban counterparts [5-8]. This is significant as the rural population in Canada in the 2011 Census was 19% of the total population [9].

To address physical inactivity and obesity, public health practitioners are increasingly looking to complement individual and family level interventions with more population-based or community level interventions targeting determinants of health to improve population health [1,10]. The built environment is one such environmental determinant of health. The built environment includes physical structures of human-made environments such as housing, schools and commercial centres; parks and public spaces; transportation infrastructure such as streets and highways, paths, and sidewalks; and neighbourhoods [11]. This study focused solely on the built environment as it relates to physical activity.

Most reviews and meta-analyses conclude that built environment characteristics and policies may encourage, provide opportunities, present barriers, or constrain physical activity, as it can influence whether a person engages in physical activity and the frequency in which they do so [12-16]: For a full review of the literature refer to [17-20]; However, much of the literature focuses on urban and suburban settings. There is a paucity of information related to the built environment in rural settings. One systematic review by Frost et al. [21] specifically addressed built environment and physical activity in rural locations by examining barriers to and motivators of physical activity among rural populations. Research in rural contexts has been repeatedly noted to be a research gap [16,22,23].

In the past decade there has been an explosion of interest regarding the potential contribution of built environments particularly land use planning and transportation, on levels of physical activity and to a lesser extent, on weight/obesity [24,25]. In the province of Ontario in Canada, the important link between the built environment and healthy communities has been recognized at a provincial level with incorporation of the built environment into the Ontario Public Health Standards (OPHS) policies.

There are 36 public health units in Ontario each serving a distinct geographic region that are individually responsible for serving the population within their borders [26]. They are staffed by public health nurses, public health inspectors, public health physicians, health promoters, dietitians and epidemiologists, typically with a medical officer of health (MOH) as head of management. The OPHS outline core functions of public health units and expectations for boards of health, which are responsible for providing public health programs and services in Ontario [27]. Public health units deliver health promotion and disease prevention programs designed to improve population health. Programs and services include: chronic diseases and injuries; family health; infectious diseases; environmental health; and emergency preparedness. Public health units are now legislated through the OPHS to incorporate the built environment into their Chronic Disease Prevention (CDP) and Environmental Health Hazard Programming [27], yet little is known about how health units who service rural settings are integrating built environment interventions into their programs.

Three reports and case studies outline some initiatives that public health agencies are involved with in the areas of land use planning and the built environment to promote healthy communities [25,28,29]. Malatest & Associates Ltd. [28] completed an environmental scan in Ontario to determine the involvement of public health agencies in the area of land use planning and the built environment [28]. Two-thirds of the twenty-eight health units that participated stated they were involved in developing policies, programs and initiatives related to land use planning and the built environment [28]. However, little detail is provided on the types of interventions being employed.

Tucs & Dempster [25] examined seven community case studies focused on promising practices to create healthier communities through community design, land use planning and planning policy development in Ontario [25]. A few of these case studies includes health unit participation.

Perrotta [29] examined how ten public health units in Ontario, three of which are rural, were working to influence land use and transportation planning processes to help create healthy and sustainable communities. The report outlined interventions and strategies employed to influence land use and transportation planning, the expertise needed to address this, and the tools and research that health unit staff require to be more effective in this field. However, none of the reports are specific to rural settings or physical activity.

The purpose of this study was to explore how rural health units in Ontario are integrating the built environment into public health interventions related to physical activity. The unique challenges addressing the built environment in rural settings and the lack of research in this area were the impetus for investigating this topic.

## Methods

This exploratory research study employed a fundamental descriptive, qualitative approach, which provides a thorough summary of events or phenomenon in everyday language and is the method of choice when straight descriptions of phenomenon are desired [30]. True to the tenets of fundamental qualitative description, the researcher did not utilize a conceptual or philosophical framework to describe the events or phenomenon [30].

This study employed two stage purposeful sampling. Of 36 health units in Ontario, 13 are considered ‘rural northern’ or ‘mainly rural’ based on Statistics Canada’s 2007 peer groups [26]. All thirteen health units were recruited. An email requesting participation was sent to each health unit’s MOH or Director. Once a response was received from the MOH, the MOH’s assistant, or Director, a recruitment e-mail was sent to the staff members within the health unit who were identified as having in-depth knowledge about the topic. They included public health practitioners and managers identified by the MOH or Director as the most knowledgeable about program planning, implementation, and policy development in relation to physical activity and the built environment. In two instances, two staff members were identified by the MOH or Director; therefore, both participants were included at the health unit’s request. A letter of consent was signed by each participant.

In-depth 60–90 minute telephone interviews were conducted by the first author and audio-taped with one exception of a participant who did not provide consent for audio-taping. A semi-structured interview guide was used (see Additional file 1). The researcher made field notes after each interview noting insights and reflections [31]. They served as a back-up in the event of audio-recording failure [32] as well as captured reflections to assist analysis.

Data collection and analysis were done concurrently. Transcripts were transcribed verbatim by the first author and a paid transcriptionist. The work conducted by the paid transcriptionist was carefully reviewed by the first author to ensure accuracy. Interviews were stripped of identifying information to protect confidentiality. Qualitative content analysis was used to analyze the data.

Using the qualitative software program NVivo9, an initial coding structure was constructed by the first author as patterns and themes were identified [33,34]. Analysis was inductive and themes were built from the bottom up. To minimize interpretation and stay as close to the data as possible, the initial coding structure was constructed using words and sentences directly from the transcripts [33]. Data was then collapsed into larger categories or ‘chunks’, moving towards broader generalizations [33,34]. Broader generalizations and themes aided in summarizing and answering the primary research questions. The final coding structure and coding for a selected set of interview transcripts were reviewed by a second researcher (RV) to increase confirmability of results. Data saturation was achieved, as no new themes or concepts emerged.

To ensure descriptive validity, member checking was conducted. It involved sending summarized results in tables to participants to ensure they accurately reflected their experiences and thoughts. Feedback provided validated the themes.

Ethics approval was received from the Faculty of Health Sciences Research Ethics Board in March 2012 prior to the commencement of the study. Ethics approval was also sought from each participating health unit’s internal review board when required.

## Results

### Participant and health unit characteristics

Twelve of thirteen health units recruited responded. Each geographic region of Ontario was represented. Two health units requested to have two participants interviewed to gain a broader perspective of interventions offered by their health unit for a total sample of 14 interviewees. Of them, seven (50%) were health promoters, four (29%) were public health nurses, and three (21%) were managers. Organizations described as: 1) serving or being a department of a single county; 2) two counties or districts; or, 3) three counties or districts were labeled upper tier governments by many respondents. Participants also noted that they worked with 2 to 24 lower tier governments, such as cities, towns, municipalities and townships.

Many respondents described the geographic area their health unit serviced as being large in land mass with large distances between destinations or points of interest. All health units served populations with less than 200,000 people with the exception of one health unit that contained a large urban population centre.

### Interventions

Participants were asked to describe how their health unit was integrating the built environment into public health interventions to address physical activity in their communities. ‘Health interventions’ was defined as public health activities, interventions, initiatives, program planning and delivery, and policies related to the built environment. A number of major themes were identified related to built environment interventions and physical activity as summarized in Table 1 and discussed below.

**Table 1:** **Summary of Themes and Subthemes Related to Interventions**

**Major themes**	**Subthemes**	**Description of subthemes**
Engagement with policy work at a county and/or municipal level	Input into official plans	• Review and comment on official plans
		• Development of policy statements for official plans
	Input into master plans	• Input into transportation master plans
		• Input into AT, cycling, pedestrian, parks, trails, and recreation master plans
	Policy document resources	• Planning Guide Resource
		• Toolkit for creating healthy communities
		• Policy statements for official plans
	Input on individual planning applications	• Input on sidewalks, walkability, design, accessibility
Building and working with community partners, committees and coalitions	Involvement with community coalitions or committees	• Participation on trails, AT, cycling, master plan, healthy communities committees/ coalitions
		• Participation in Healthy Communities Partnership
	A resource for community groups or committees	• Leadership role (coordinator, co-chair, advisory role)
		• Research and evaluation (provides evidence, best practice information, health status information)
		• Building capacity (Provides training to partners, builds linkages between partners)
Gathering and providing evidence	Research or data collection on BE characteristics	• Qualitative data collection regarding community perceptions and attitudes regarding the BE and AT values, needs, barriers and concerns
		• Walkability and bikeability assessments
	Research or data collection on PA levels or modes of AT	• PA levels, type of activity, frequency and duration
Program development and implementation	Events held in the community	• Car Free or Open Street events
		• Events to highlight existing AT infrastructure
		• Biking events (i.e. Bike to Work Week)
	Resource development and dissemination	• Develop or promote guides for trails, bike routes
	Comprehensive community based programs	• Share the Road program
		• Ontario Communities walkON initiative
		• School Travel Planning Projects
	Hosting knowledge sharing opportunities	• Participatory walkability and cycling workshops and conferences, AT workshops, healthy cities workshops for the community, municipal decision-makers and public health staff
Social marketing, information sharing, and awareness raising	Awareness raising and information sharing: For the community, municipal decision-makers, and public health staff	• Presentations on specific programs
		• Campaigns to raise awareness on the BE, PA and AT
		• Social marketing regarding the BE, PA and AT
		• Deputation to Councils
	Promotion of AT and trails	• Trail promotion
		• Promote walking, biking and AT

Note. AT = active transportation; BE = built environment; PA = physical activity.

#### Theme 1: Engagement with policy work at a county or municipal level

The primary theme that emerged was policy related activities. All health units reported to be influencing or developing policy at a city, county and/or municipal level, depending on the political structure and organization of the region served by the health unit. Respondents indicated their health units participated on review or steering committees that influenced, reviewed and/or commented on official plans and master plans including transportation; active transportation; cycling; parks and trails; and recreation. Many participants noted formal procedures, such as circulating plans through various public health departments and providing feedback on issues such as sidewalk, road and trail connectivity, land use planning, park space, and active transportation (cycling, pedestrian) planning. A participant described this work:

*I’ve been involved in reviewing the Official Plans and Master Plans as they come forward… things we would look at is sidewalk connectivity, trails within the new development. Are there any bike lanes proposed, if not, why? Could we propose some? So we’re really looking for that connectivity, and land use planning, park space, issues such as that* [Participant 7].

Another participant described the health units’ input and incorporation of active transportation into a Master Plan:

*So there has been a commitment of money to create infrastructure when repaving roads, to add a paved shoulder of 1.5 meters. And signage…So we provide input and have created a strategy to identify priority routes* [Participant 12].

A few health units developed resources to assist with policy related activities, such as a toolkit for creating healthy communities, a planning guide resource for planning and policy decisions, and a healthy community design guide with policy statements for official plans. One respondent described a planning guide:

*The guide outlines a wide range of possible decisions municipalities and politicians face when making planning and policy decisions regarding active transportation* [Participant 8].

A few participants discussed involvement in reviewing individual planning applications for input on issues such as walkability, design issues, and accessibility to encourage physical activity and active transportation, for example:

*…let’s say a subdivision or a Walmart going in somewhere… we’ve been providing specific comment(s) on specific places with recommendations around things like increased sidewalks, and the frontage of the stores and etcetera to increase the desire to walk and that sort of thing* [Participant 2].

Engagement in policy work was done in partnership with others, which relates to the next theme.

#### Theme 2: Working with community partners, committees and coalitions

Working with community partners, committees and coalitions was a primary activity by all participants. Each respondent discussed involvement in groups, such as trails committees, cycling coalitions, active transportation committees, and transportation working groups. Half of the interviewees were involved in a local Healthy Communities Partnership (HCP), a provincially funded program that provides organizations, community members and partners an opportunity to co-create healthy, active communities. Some respondents noted that their HCP identified the built environment, physical activity and/or active transportation as key community priorities.

Health unit staff functioned as a resource for community groups and committees, and/or served leadership roles, such as chairing committees. Others were knowledge brokers providing evidence on the relationship between the built environment and health, sharing best practice information, and providing local health status information. Lastly, a couple of respondents helped build community capacity by training community partners and municipal leaders on built environment impacts on health, physical activity and active transportation, and facilitating community partners linkages.

The following excerpt summarizes public health staff functions in partnerships:

*We sit on community committees…[]. We view one of our key roles as being able to share best practice information, health status information, information on the connection between the built environment and health. One of the key things that we do also is we help to connect partners together… [] We have a really good sense of who all the partners are out there and were able to connect those partners together* [Participant 5].

The development and strengthening of relationships with non-traditional partners, such as engineers and planners, and improved collaboration were stated as key developments from interventions. Further to the knowledge broker role, is the need to gather and provide evidence— the next theme.

#### Theme 3: Gathering and providing evidence

The majority of respondents remarked that evaluations of interventions were being done either informally or not at all. Reasons for the latter were lack of human and fiscal resources; lack of skills; and difficulty evaluating chronic disease prevention programs due to complexities such as multiple program components, differing contexts, and long-term outcomes.

However, all health units were engaged in gathering and providing evidence on built environment characteristics or physical activity levels through research projects, community needs assessments, and local data collection.

Data collected on built environment characteristics was subjective based on community perceptions and attitudes. Many surveys were conducted with community members and municipal staff regarding needs, perceived barriers to active transportation, and concerns regarding built environment characteristics. PhotoVoice, a research method utilizing photography to capture individual perspectives [35] was carried out at one health unit which resulted in policy changes:

*The [PhotoVoice project] was with grade five students and it was all around pedestrian safety, what helps you or hinders you from getting to school safely… Another PhotoVoice project… it was just what keeps you safe and healthy in your community, or what makes you unsafe, unhealthy in your community. And some of the results there also showed a lot of design issues; accessibility issues, like curbing, just sidewalks in poor repair…And the results of that PhotoVoice project were actually instrumental in getting some of the language changed in our official plans to support active transportation* [Participant 14].

Almost half of respondents discussed objective, quantitative data collected on built environment characteristics such as conducting walkability assessments or GIS mapping to observe sidewalk existence, connectivity, and active transportation priority routes and safety. One participant noted, “*We use a walkability checklist…mainly to engage planners and people at the municipal level…to increase their awareness”* [Participant 12].

Most physical activity data collection was from external sources (e.g., CCHS). Internal data on physical activity (type, frequency, duration) was obtained through self-reported surveys and community assessments. One health unit collected data on physical activity levels by means of completing pedestrian and cycling observation counts prior to- and after changes were made to the built environment. Such evidence could be used for program planning – the next theme.

#### Theme 4: Program development and implementation

Many respondents described program development and/or implementation of initiatives or events to engage people on built environment (e.g., Open Street events, where streets were closed to vehicular traffic). Others organized group walks and runs to promote trails and existing infrastructure encouraging physical activity. Many biking events were developed, such as Bike to Work Week, commuter challenges, cycling festivals, and guided cycling tours. Many respondents developed and/or promoted resources such as guides for trails and bike routes, highlighting the connections between communities and identifying priority areas of work for development.

Half of the respondents discussed school programs, such as Active and Safe Routes to School and School Travel Planning Projects. These were often multi-faceted and included activities such as: community walkabouts to identify existing active transportation infrastructure; surveys to identify active transportation barriers; and promoting walk or wheel to school days.

Most health units hosted community workshops, public forums, and conferences on walkability and cycling, active transportation and/or on creating healthy cities. Many of these events intentionally involved decision-makers and stakeholders or were tailored specifically towards elected officials or municipal staff. As noted by one respondent:

*…we strategically made it so that it was only engineers in this forum and road personnel so that they felt really open to having open discussions and it wasn’t with other disciplines…now we have their buy-in* [Participant 9].

A way to get community buy in is through social marketing and raising awareness- the final theme.

#### Theme 5: Social marketing and raising awareness

Almost all respondents discussed awareness raising activities achieved primarily through presentations to schools, community groups, elected officials, county/municipal staff, and the public regarding ways the built environment can impact health and physical activity. Representation at public meetings and deputations to Councils to engage local decision-makers were also noted. One respondent noted how awareness raising activities had changed a notable community champion’s perception:

Many participants used social marketing techniques to increase general awareness or to promote campaigns as the example shows:

*…[the committee chair] lives in a neighbourhood that has no sidewalks and she said she recalls a number of years ago when her street was being resurfaced, the petition circled in her neighbourhood, no sidewalks… “I signed it without thinking of the greater implications… Look at me now.” So I mean that mind shift can happen right* [Participant 3].

*We also do a lot of awareness raising through the local media—and having newspaper articles on various topics related to being active and healthy communities, that sort of thing. I do a once a month radio interview on our local community radio station and talk about various aspects of physical activity and health and healthy communities* [Participant 10].

### Results based on differences in rural contexts

Themes were analyzed for differences between health units based on rurality, determined based on: population density of the population served; and percentage of the population that lived in rural areas. Parameters used to compare health units included: health units that served a population density less than and greater than 20 people per square kilometre. Rural area population parameters were health units that served a rural population greater than 50% of the population and those that had less than 50%. There were no differences in interventions used to address built environment by population density or percentage of the population that was rural.

## Discussion

This study demonstrated that there is much work being conducted in health units serving rural communities in Ontario.

Participants focused a lot of their discussion around two highly interconnected themes: policy related activities and establishing and working with community partners. Policy work was often done in collaboration with community committees and coalitions and involved the public health sector working with non-traditional partners, such as municipal planners and engineers. It also involved reviewing and providing feedback on official and master plans regarding active transportation and planning and design of healthy, sustainable communities. Historically, public health practitioners and planners collaborated in the 19^th^ century to address public health concerns, such as infectious diseases associated with overcrowding and poor living conditions [11,18]. Many researchers and policy makers have called on public health officials to reconnect and work with land-use planners, builders and engineers to address the built environment, particularly around physical activity and active communities, to ensure that health impacts are considered when planning and making decisions about the built environment [11,16,36]. This study clearly indicates that the relationship between public health and planning is being reestablished, as planners and public health professionals now partner in designing healthy communities to address chronic disease prevention and risk factors such as obesity and physical inactivity. The role of public health has become clearer for planners, engineers, municipalities and counties, as public health’s input has been sought for local planning decisions related to healthy communities. Formal mechanisms to improve intersectoral collaboration include: creating cross-sector committees to address community priorities; interprofessional conference attendance, and collaborative training sessions with public health practitioners and planners.

Recommendations have been made for public health practitioners to advocate for or participate in local planning processes to support and/or contribute to policies, master plans, smart growth principles, and planning and zoning meetings to create healthier environments [13,18,37]. This has seemingly struck a chord, as the current study found health unit policy activity participation was primarily at the county and/or municipal level. The current study indicates public health and community partners are participating in the policy process through planning. Planners and public health staff are also collaborating successfully in rural areas. All respondents discussed their health unit’s involvement in influencing municipal or regional planning policies from a public health perspective. Participating in the development of local strategic policy documents enables health agencies to respond to local community needs and their unique contexts. However, no examples were found in the literature evaluating the effectiveness of public health’s input into municipal policies, such as planning documents. Public health input is being sought by municipalities, but in the absence of long term evaluation, the effectiveness of this input and whether this has improved health outcomes is unknown. Evaluation is needed to determine whether input into and participation in this process has measurable changes to municipal plans themselves and whether these changes translate into any long term health outcomes.

The Rural Communities Impacting Policy project found people living in rural communities are often excluded from policy decisions [38,39]. Dukeshire & Thurlow [38] reported rural communities face significant challenges in the policy arena such as: lack of understanding of the policy process; lack of community resources, education and training; and lack of access to information such as research. Further, Aytur et al. [40] found public health practitioners were less involved in rural planning processes than urban ones. However, the current study suggests public health professionals and community partners are participating in the policy process as it relates to planning documents in rural settings. The current study did not compare urban with rural health units, but examined the degree of rurality of participating rural health units. It would be valuable to compare urban and rural health units in Ontario to determine the extent to which they are participating in the policy process, if the above mentioned challenges exist in more urban settings, and to determine the successes and challenges regarding the policy process.

Participants discussed the need for information sharing among health units serving rural populations such as how-to documents and tools for practitioners to contribute to planning documents, such as standardized templates for reviewing planning applications and policy documents. A locally driven collaborative project titled, “*Building Rural Health Communities Research Project”*, is underway to identify evidence-informed strategies and models of practice for land use planning policies, procedures and designs for the built environment to improve population health outcomes . The aim is to develop a toolkit to advise public health professionals, land use planners, municipal staff and elected officials of effective strategies and models of practice.

The importance of developing partnerships and multi-sectoral collaboratives has been highlighted repeatedly [11,16,29]. Working in collaboration with community partners, networks, governmental bodies both with and outside the health sector is a foundational principle in the OPHS [27]. Hence, it is not surprising that all participants highlighted working with partners. In rural settings, with large geographic distances between points of interest and fewer resources (human and financial), the need for more comprehensive, collaborative efforts may be even more important. Aytur et al. [40] found planners were more likely to collaborate on active transportation plans with more non-traditional interest groups, such as business groups and non-profit groups in rural settings than when developing urban plans. However, public health staff were less likely to be involved with planners in rural settings. The authors indicated there was greater collaboration and partnership building in rural settings but less public health involvement [40]. Contrary to this work, the current study found public health staff were collaborating with many non-traditional partners, such as planners, on many interventions including transportation plans. This may be due to the public health shift towards more policy development activities. The OPHS have established requirements for fundamental public health programs and services, which includes health promotion and policy development [27]. Half of the participants in the current study mentioned the OPHS as influencing their health unit in addressing the built environment.

The theme- gathering and providing evidence- confirms the need for evidence-based decision-making during program development and implementation. Participants acted as knowledge brokers providing evidence on the relationship between the built environment and health and local health status information. Most evidence was subjective in nature and based on community perceptions, which is important as perceived safety of one’s environment can play a major role in the decision to engage in active transport [8,18]. Data collection at the health unit level that is based on community perceptions may be of great significance in addressing local physical activity levels. Many studies have examined the perceived environment and found personal preferences and perceptions can impact active transportation and physical activity [19,23,41]. Many participants noted that there were little to no evaluations being done of interventions coinciding with the work by Dunn [19] who addresses complexities of evaluating built environment interventions in population health.

Although many interventions are being employed across Ontario to address built environment and physical activity, it is likely that comprehensive programming – including a combination of interventions – will be more successful than any one intervention alone. For example, approaches should combine health education and awareness raising to increase community support, as well as healthy public policies. Pucher, Dill & Handy [42] summarized international case studies where comprehensive interventions in cities were successful at promoting active transportation. They included a wide range of policy interventions, infrastructure changes and marketing campaigns. The urban case studies indicate that individual interventions to promote cycling are effective, but substantial increases in bicycling require multi-faceted packages of many different and complementary approaches. The current study identified a myriad of interventions are being employed in rural settings such as: developing and implementing supportive policies around planning and design of healthy communities; partnership building, particularly intersectorally; gathering evidence on associations between built environments and physical activity levels; and social marketing campaigns. However, the current study did not evaluate the effectiveness of individual interventions or which combination of interventions would be most effective to improve physical activity levels. Future research is needed to explore complex multi-component interventions.

A number of limitations of this study are important to point out. As noted by many participants and supported by Dunn [19], the importance of local context must be emphasized in studying public health interventions. The degree of rurality of each region in this study varied greatly. Thus the term ‘rural’ should not be used as a ‘catch all’ term. Interview questions in this study did not probe for differences in interventions with respect to these rural contexts. This study did not expose variability well and, therefore, limited analysis explored inter-rural differences. This is a gap found in the literature as well. Although there were no clear differences seen based on governance, geography (land mass), and population density in this study, future researchers should identify what constitutes variations of ‘rural’, and investigate what role these characteristics have on success of built environment interventions.

Also, limiting interviews to key informants from each health unit may have influenced the depth of data collected. Many health units service large geographic regions and respondents were only able to speak to knowledge of their specific community particularly where numerous satellite offices or multiple counties and/or districts existed. Focus groups may have provided more comprehensive representation of interventions being employed. Also, it was not always clear if interventions were occurring in more populated rural regions versus hamlets or villages. Further probing would have provided more contextual information that would have assisted transferability of findings and assisted in analyzing for inter-rural differences. Conducting case studies could overcome this limitation.

Research results were shared with participants in a research summary and fact sheets tailored to managers and practitioners. Participants were encouraged to share this information with colleagues, particularly within CDP or other relevant programs. Additionally results were communicated with and distributed to professional groups, including the Ontario Public Health Associations Built Environment Working Group and the project coordinator of the Public Health Ontario Locally Driven Collaborative Project on rural built environment. The latter is a rural best practices project which aims to fill an identified gap regarding best practices in these settings.

Despite these limitations, results highlight the breadth of work being conducted in rural Ontario that address the built environment and physical activity. They help to inform public health practitioners, managers, decision-makers, and policy-makers about the multiple ways to contribute to comprehensive built environment activities that include a mix of: policy work, intra and intersectoral partnerships, community assessments, program development and implementation and social marketing within their rural environments.

## Conclusion

This research utilized a descriptive qualitative approach to explore how rural public health units in Ontario are integrating built environment interventions related to physical activity. Health unit staff have been employing a variety of activities to address the built environment to enhance physical including: engagement with policy work at a municipal level; building and working with community partners, committees and coalitions; gathering and providing evidence; developing and implementing programs development; enhancing social marketing and awareness raising. Evaluation of interventions to date has been limited.

This research is both timely and relevant as evidenced by the explosion of research in the past five years on built environment and its effects on physical activity and the inclusion of the built environment in recent public health policy in Ontario. Future research is encouraged to explore if and how public health actions towards the built environment differ between rural and urban settings. In addition, research should identify what constitutes variations of ‘rural’, and investigate what role these characteristics have on success of built environment interventions.

## Additional file

Additional file 1: **Interview Guide for Semi-Structured Interview.**

## Abbreviations

CCHS: Canadian community health survey
OPHS: Ontario public health standards
CDP: Chronic disease prevention
MOH: Medical officer of health
