# Supplementary material for: Built environment interventions aimed at improving physical activity levels in rural Ontario health units: a descriptive qualitative study
Source: BMC Public Health. 2015 May 3;15:464. doi: 10.1186/s12889-015-1786-2 (PMC4426164; doi:10.1186/s12889-015-1786-2)
Supplement: Supplementary file 1 — Interview Guide for Semi-Structured Interview. [file 12889_2015_1786_MOESM1_ESM.docx]

**Additional file 1: Interview Guide for Semi-Structured Interview**

I’d like to thank you for taking the time to be interviewed today. Before we begin, I’d like to remind you that the interview is voluntary and if there are any questions that you don’t feel comfortable answering or that you do not feel that you know the answer to, please let me know and we go on to the next question. I will ask you to state your name for the purpose of transcription only. You will be assigned a numerical code for your interview to protect your confidentiality. However, due to the small number of rural health units and participants involved in this study, it may be difficult to protect your identity. Others may be able to identify you on the basis of references you make. Every precaution will be taken to ensure that this risk is minimized.

For the purposes of this study, ‘interventions’ will refer to any public health work, activities, interventions, initiatives, program planning and delivery, and policies related to the built environment.

Study questions:

1. Please briefly state your name, your discipline, your position and what roles or tasks you fulfill within your health unit?
2. Please briefly describe the community that you serve.
3. What does the term built environment mean to you?
4. What program areas within the health unit address the built environment?
5. Who are the individuals involved in these program areas?
6. What is your role in relation to the built environment at your health unit?
7. What, from your perspective, are the most important impacts of the built environment on population health?
8. What public health interventions have been/are being/will be employed to address the built environment to promote a healthy community?
9. What **influenced** your health unit`s decision to implement these strategies or interventions?
10. Has your health unit evaluated any of these interventions?

If so, how was this evaluation done?

1. For successful interventions addressing the built environment and physical activity, what do you feel contributed to their success?
2. Are there any local contextual issues that may impact your health unit’s ability to address the built environment?
3. This is a rural area. Does this bring any special challenges?

Does this provide you with any opportunities?

1. What type of local or neighbourhood data are you collecting regarding built environment characteristics or physical activity levels?
2. What enablers have your health unit encountered when developing and implementing interventions to the built environment and physical activity?

What lessons were learned?

1. What barriers has your health unit encountered when developing and implementing interventions related to the built environment and physical activity?

What lessons were learned?

1. What is the relationship or level of involvement between your health unit and:
   1. the local planning department in your area;
   2. the local transportation department in your area;
   3. the engineering/public works department in your area?

Please describe.

1. What types of collaborations/partnerships exist between your health unit and community agencies/organizations regarding healthy, sustainable communities and the built environment?

In what ways do you partner with community agencies/organizations?

1. What would assist you or your health unit in your work related to enhancing the built environment to promote physical activity?

**THANK YOU FOR YOUR TIME AND COOPERATION**
